# Spontaneous Severe Haemoperitoneum in the Third Trimester Leading to Intrauterine Death: Case Report

**DOI:** 10.1155/2011/173097

**Published:** 2011-09-14

**Authors:** Harriet Williamson, Radha Indusekhar, Alexander Clark, Ismail M. Hassan

**Affiliations:** ^1^Keele University Medical School, Keele ST5 5 BG, UK; ^2^University Hospital of North Staffordshire NHS Trust, Stoke on Trent ST4 6QG, UK

## Abstract

Spontaneous haemoperitoneum during pregnancy is a rare but potentially catastrophic cause of acute abdominal pain. A healthy 37-year-old primigravida presented with acute abdominal pain and hypovolaemic shock at 37-weeks gestation. An emergency caesarean section was indicated on the clinical suspicion of placental abruption. However, an ultrasound scan confirmed the absence of a fetal heartbeat, and, in light of the mother's haemodynamic stability, a vaginal delivery was deemed most appropriate. Subsequent imaging, due to deterioration over the following 24-hours, revealed a large heterogenous haematoma within the pelvic cavity, which was later found to be caused by severe pelvic endometriosis. Despite fertility problems associated with severe endometriosis, advanced assisted reproductive technology enables more of these patients to become pregnant, highlighting the need to be aware of this rare complication in pregnancy.

## 1. Introduction

Spontaneous haemoperitoneum during pregnancy is a rare complication but can be life threatening for both mother and foetus [1]. This complication can occur at any gestational age; however, the majority of cases develop in the third trimester. Ginsburg et al. reported that 61% of cases of unprovoked peritoneal bleeding occur antenatally, 19% intrapartum, and 21% puerperal [2]. In 1950, a maternal mortality rate of 49% [3] was reported; however, subsequent advances in resuscitative, anaesthetic, and operative techniques have resulted in a decline in this figure to 3.6% [2]. Perinatal mortality due to spontaneous haemoperitoneum has remained high at 31%–36% [2, 4]. We report the case of a 37-year-old woman with spontaneous intra-abdominal bleeding accompanied by foetal death *in utero* at 37-week gestation, despite prompt admission. 

## 2. Case Report

A 37-year-old woman, gravida 2 para 0, presented at 37-week gestation with severe acute abdominal pain which had commenced two hours prior to admission. Prior obstetric history included subfertility problems for two years; however, this current pregnancy was achieved naturally. An antenatal ultrasound scan revealed a left haemorrhagic ovarian cyst measuring 6  × 6  × 5 cm at 7-week gestation. An abnormal oral glucose tolerance test (OGTT) at 28-week diagnosed gestational diabetes which was diet controlled. 

She appeared acutely ill with a body temperature of 35.8°C, blood pressure (BP) of 60/40 mmHg, pulse rate of 112 bpm, respiratory rate of 16/min, and oxygen saturation of 97% on air. She did not report rupture of the membranes, drug abuse, recent intercourse, and abdominal pain, and there was minimal vaginal bleeding. Physical examination on admission concluded hypotensive shock; therefore, fluid administration was commenced. On examination, the abdomen was soft and lax with no tenderness. Pelvic examination revealed a soft cervix with dilation of 2-3 cm and no contractions. Artificial rupture of the membranes (ARM) was conducted showing a slightly blood-stained liquor which led to the suspicion of a placental abruption. 

External foetal auscultation was queried. Therefore, the patient was rushed to theatre for an emergency Caesarean section (CS); however, beforehand an ultrasound scan concluded intrauterine foetal death. Initial laboratory studies on admission revealed haemoglobin (Hb) of 10.9 g/dL, platelet count of 366  × 10^9^/L, and normal INR and fibrinogen levels. A catheter urine dipstick revealed proteinuria of 3+. The patient was resuscitated with 3 L of Hartmann's solution and 2 units of O negative blood. A CS was no longer required because the patient became haemodynamically stable after fluid resuscitation with a BP of approximately 140/95 mmHg and a pulse rate of 106 bpm. Instead, the patient was transferred to the High Dependency Unit (HDU) where she remained stable. Therefore, a vaginal delivery was considered to be the most effective course of action. She was augmented with Syntocinon. 

After a 15-hour labour, she delivered spontaneously a 2.7 Kg female foetus. The total blood loss was 450 mL including retroplacental clots of 150 mls. The postmortem later revealed that there were evidential features of hyperacute hypoxia; however, uniquely the placenta was normal. Postnatally, she looked well with a BP of 130/90 mmHg and minimal blood loss. The patient remained in the delivery suite for further observations. 

She remained stable over the next 24 hours with a BP of 120/75 mmHg, pulse rate of 100–115 bpm, an adequate urinary output of 30–340 mls/hr, and normal involuting uterus with no undue vaginal loss or abdominal pain. However, her Hb dropped to 7.2 gm/dL and a bedside ultrasound scan revealed a left-sided broad ligament haematoma measuring 6  × 8  × 9 cm with an empty endometrial cavity. As she was haemodynamically stable, conservative management seemed to be a suitable option with transfusion of 2 units of packed red blood cells. This led to stabilisation of the Hb level to 8.9 g/dL. 

By 48 hours after delivery, it was felt that the patient's clinical course did not correlate with the diagnosis of placental abruption. Therefore, an intravenous and oral contrast computerised tomography (CT) scan was requested. CT scan was reported as demonstrating a large heterogeneous haematoma within the pelvic cavity lying laterally to the left of the uterus and extending cranially to the level of the inferior pole of the left kidney. The bulky uterus was unremarkably consistent with postpartum status. There was suggestion of a small area of focal haemorrhage within the haematoma. As the patient was still haemodynamically stable, conservative management was continued with the addition of a broad-spectrum antibiotic. After an uneventful recovery period, she was discharged home within one week of delivery.

**Figure 1:**
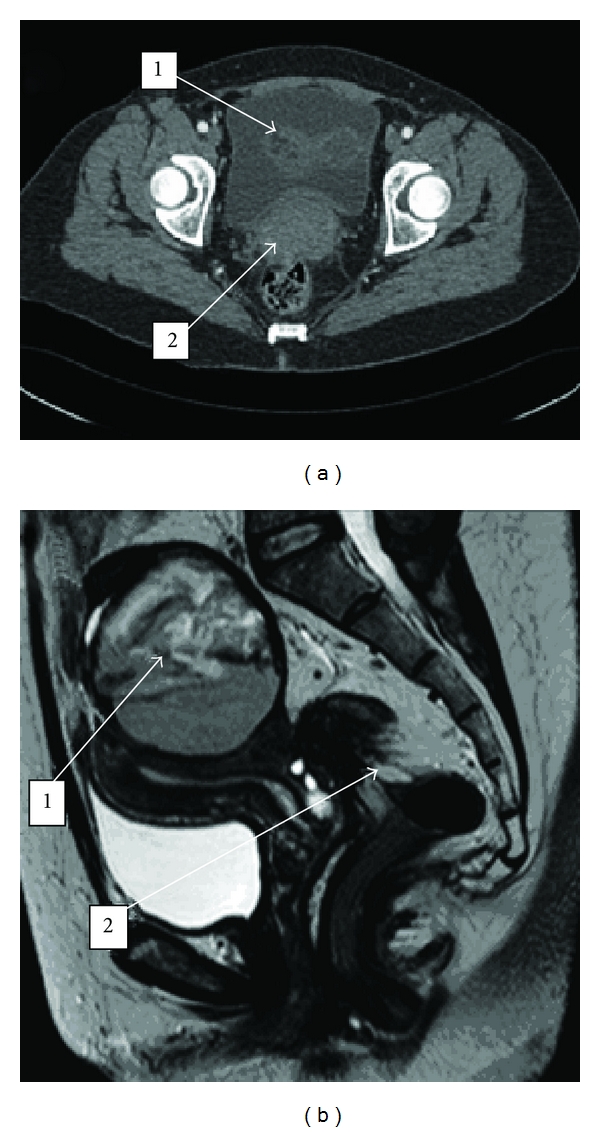
(a) An axial CT angiogram of the pelvis performed 3 months after delivery. (1) indicates a pelvic mass. (2) demonstrates a bulky cervix with a surrounding ill-defined outline where an underlying cervical malignancy could not be ruled out at this point. (b) T2 sagittal MRI scan taken 4 months after delivery. (1) highlights pelvic endometriosis with a left endometrioma sitting over the uterine fundus. (2) demonstrates tethering of the plaque to the ventral rectosigmoid junction indicating the severity of the endometriosis.

Subsequent investigations were required to aid in the confirmation of a diagnosis. An ultrasound scan a week after discharge revealed a haematoma to the left side of the uterus measuring 8  × 8  × 6 cm. A CT angiogram scan 3 months after discharge showed a well-defined soft tissue mass in the pelvis measuring 10  × 8  × 9 cm, representative of a left adnexal lesion with an abnormal and enlarged cervix with an associated left hydro-utero-nephrosis. This well-defined, large lesion was suspected to be a complex or complicated ovarian cyst rather than a haematoma (Figure 1(a)). An MRI scan was subsequently conducted 4 months after discharge to further characterize this mass. MRI scans revealed possible pelvic endometriosis with a large left endometrioma measuring 9  × 7  × 7 cm (Figure 1(b)). A parametrial endometriotic plaque was situated at the junction between the lower uterine segment and the cervix with tethering of the ventral rectosigmoid colon. Therefore, the concluding imaging diagnosis was severe pelvic endometriosis. 

## 3. Discussion

Spontaneous haemoperitoneum during pregnancy is rare, though it has life-threatening complications [2]. The typical presentation of this complication is sudden onset abdominal pain with signs of hypovolaemic shock without revealed bleeding. A marked reduction in Hb is a frequent finding [2]. The diagnosis is rarely made before exploratory laparotomy; therefore, a misdiagnosis of placental abruption is frequently found. 

One of the common reasons for the spontaneous haemoperitoneum is rupture of utero-ovarian vessels. The aetiology of this condition is poorly understood. Hodgkinson and Christensen have hypothesised that the possible cause is dilated utero-ovarian vessels. This can result from the increased physiological demands of pregnancy and various muscular activities, such as coughing, defaecation, coitus or straining during the second stage of labour, which all exclusively result in a sudden rise in venous pressure. In a healthy pregnant women, extensive physiologic hypertrophy of the uterine vessels deals effectively with pressure fluctuations [3]. Therefore, the possible existence of additional vascular defects is suspected; a possible origin of this could be decidualised endometriosis invading the utero-ovarian vessel wall [5]. 

Our case reported a suggested diagnosis of endometriosis three months postpartum. Endometriosis affects approximately 10% of women of reproductive age generally involving the peritoneum, ovaries, and rectovaginal septum with symptoms of dysmenorrhoea, pelvic pain, deep dyspareunia, and infertility [6]. 

Pregnancy can have beneficial effects on endometriosis by promoting involution of the endometrial implants, and it has recently been suggested that there is a protective role against preeclampsia [6]. However, rare significant endometriosis-related complications can occur late in pregnancy in the form of spontaneous haemoperitoneum [4]. This is either a result of endometriosis invading the utero-ovarian vessels or bleeding from the endometriosis implants themselves. Chronic inflammation caused by endometriosis may make the vessels more fragile and therefore more prone to rupture. Resultant adhesions may produce greater tension on the utero-ovarian vessels especially when the uterus enlarges during pregnancy. Furthermore, severe endometriosis during pregnancy could invade surrounding areas resulting in spontaneous haemoperitoneum or decidualised endometriosis may cause utero-ovarian vessel perforation [7]. 

Decidualisation occurs with the differentiation of mesenchymal cells dependent upon sustained progesterone levels. Recent evidence has suggested that endometriosis could become progesterone resistant; a decrease in progesterone levels in the latter stages of pregnancy is associated with a reverse in the decidual phenotype and increased expression of inflammatory cells, proteolytic breakdown of the extracellular matrix, cell death, and peritoneal bleeding [8]. 

The patient in this paper presented with acute abdominal pain and intrauterine foetal death. An emergency CS was not indicated at the time because the mother was haemodynamically stable; consequently, a vaginal delivery was performed. However, 12 hours after delivery, the patient's Hb dropped to 7.2 gm/dL despite the patient remaining haemodynamically stable. Although an ultrasound scan diagnosed a left broad ligament haematoma, the clinical examination did not correlate with this diagnosis; therefore, a CT scan was requested 48 hours after delivery. This scan showed generalised bleeding with localised small focal extravasations of contrast within the haematoma, but the origin of this bleeding could not be identified. As she remained stable, conservative management seemed a suitable option. Further imaging was requested after resolution of the haematoma, to look for any underlying vascular malformations to explain the cause of the reported haematoma. The CT scan identified the pelvic mass whilst the MRI scan suggested pelvic endometriosis. Therefore, the diagnosis of endometriosis was made after the delivery even though an early antenatal scan revealed a left ovarian haemorrhagic cyst/endometrioma.

Maternal mortality rates due to spontaneous haemoperitoneum have declined due to subsequent advances in resuscitative, anaesthetic, and operative techniques; however, despite this the foetal mortality has remained constant at a rate of 31% [2], probably because these patients present so late. The most important factors determining foetal outcome are the degree of prematurity and the severity of haemoperitoneum. 

An unknown diagnosis of endometriosis prior to this pregnancy meant that the initial management was very challenging. It would be beneficial to identify and diagnose women with endometriosis prior to pregnancy to ensure closer antenatal followups.

## 4. Conclusion

Endometriosis affects approximately 10% of women of reproductive age and is a major known cause of infertility. With an increasing number of patients with endometriosis becoming pregnant, obstetricians must be aware of this rare cause of acute abdominal pain in pregnancy so that close antenatal followup and prompt intervention is employed.
